# Misfiring

**Published:** 1909-10-09

**Authors:** 


### Motoring Notes.

### MISFIRING.

The chief complaint from which engines suffer is the fault of missing fire. The cause is often very obscure and difficult to diagnose. Sometimes the trouble disappears of itself, and at others defies all one's efforts to remedy. A fruitful cause of irregular ignition is weakness of the accumulator, so when it occurs you should begin at that end of the electrical gear and satisfy yourself that all is well with the battery. Next have a look at the other end—the plugs—and then go over the ground between. See if the contact blades rub firmly on the cam. See if the tremblers on the coil are vibrating perfectly. You will find out by opening the compression taps and turning the engine slowly. Perhaps you will discover that one trembler is only fluttering. You remove the contact screw and find it badly pitted. The rivet on the blade has a minute point fused on it, just opposite the part of the screw that has become pitted. With a few strokes of a smooth file and a little adjustment of the contact screw you are rewarded with a buzz that can be heard some distance away. It may be a high-tension wire that has touched the exhaust pipe and lost its insulation, or a low-tension wire that has chafed against a water-pipe, or a loose terminal somewhere perhaps on the contact breaker. Sometimes the earth return wire breaks where it is fastened to the engine or to the frame, and then the misfiring will occur intermittently. A wipe contact should have ample means of return. The film of oil on the bearings of the second speed shaft, and the other resistances between the little brass inset and the frame of the car, are almost certain to interfere with a free return, so it is better to provide some other means of return, such as a wire attached to the plate on which the blades are mounted and connected to a nut on the engine or frame. A good plan is to fix a supplementary blade to rub on the centre of the fibre cam so as to make contact on the shaft on which the cam is fixed, and earth the wire from this blade.

If you are sure the ignition is all right, and still the misfiring persists, suspect the carburettor. A partly blocked petrol pipe will produce the symptoms, so remove the nut which connects the petrol pipe with the carburettor, and try blowing back into the tank with the tyre pump. If this rough and ready method fails, disconnect the pipe altogether, and see if it is clear. The pump will clear it if you can make a joint of some sort, either by removing the valve connection or using a bit of rubber tube, or even wrapping a piece of rubber strip round and wiring it in place. The obstruction may be in the narrow orifice below the needle valve; you can find out by dismantling the carburettor. There may be a particle of dust in the spray nozzle, which is carried up now and then to the tiny hole for delivering the petrol jet, and occasionally falls back to the bottom of the passage. Clean the jet out thoroughly, and wash through the passages with petrol before refitting the parts, when no further trouble should be experienced on the road.

### The Necessity of Attention to Small Details.

" My engine ran well for the first few months I had the car, but now I am having all kinds of trouble with it. Sometimes it won't run at all." Such is the tenor of more or less frequent complaints received by manufacturers from one end of the year to the other, as has been remarked by a well-known motoring writer in an American magazine. These tales of woe generally differ in detail, but in principle all of them spring from the same fount, all have practically the same basis, and all receive the same treatment from the maker, who through lengthy experience with this class of owner knows just how to deal with him. Probably in less than ten per cent. of such cases, all told, is the complaint really one with its foundation based upon an actual defect in the engine.

With few exceptions, the general run of drivers who are loudest in their protests against their motors would have considerable trouble in running anything, even if that were nothing more complicated than the domestic sewing machine. Keeping the engine clean, seeing that its bearings and different parts are kept in proper adjustment, attending to the tightening of nuts subject to vibration, keeping up the water and lubricating oil supply, all these are regarded in the light of trouble; a good engine should run well and smoothly without the necessity of fussing over it continually. Consequently seldom more than a glance is necessary at the engine of the car of such an owner to diagnose its disease; the symptoms stand visible all over it, and over the rest of the car for that matter. They inevitably spell neglect, and are usually aggravated by abuse.

It is not uncommon for one of these complainers to go out of his way to call attention to the fact that another engine of the same make owned by someone in his neighbourhood never gives any trouble, and if not directly, at least by influence, the maker is accused of having sold a defective engine. But contrast this owner with that whom he refers to as having received a good engine in the first place, and usually regarded as being " lucky " with it thereafter. There never seems to be any trouble with the motor of such an owner; everything is always trim and neat; it always starts when required, and runs as long as it is needed without any of the breakdowns on the road with which the unfortunate one thinks himself cursed through the carelessness of the manufacturer. His opinion is that his neighbour has a better motor, regardless of the fact that it may be of the same model and date as his own. " It doesn't need such constant attention." This driver sooner or later reaches the inevitable conclusion that he has bought the worst specimen of an engine ever built, while his more fortunate neighbour succeeded in getting the best. " Viator."

